# Regional and international collaboration: key to quality research in south Asia

**DOI:** 10.1016/j.lansea.2023.100160

**Published:** 2023-02-06

**Authors:** The Lancet Regional Health – Southeast Asia

The recently published Annual G20 Scorecard–Research Performance 2022 report, by the Institute for Scientific Information, has brought the spotlight back on quality and output of research among several of the G20 group of countries. The past 3 years of the COVID-19 pandemic have markedly changed the way we conduct and share research. Most countries now understand the role of science as an evidence base for policy, practice, and communication to benefit the population and service providers. To deal with future challenges and develop economically and socially, countries now realise the need for a strong research infrastructure, uninterrupted funding to support infrastructure, autonomy in developing projects, adequate resources for training future research capacity and, most importantly, regional, and international collaborations. Coming together through G20 is a good opportunity for nations to leverage lessons learnt from the pandemic and look towards a stronger future built on evidence-based collaborative research.

India and Indonesia are the only countries from WHO South-East Asia region to feature in the G20 group. India ranks top among Asian countries in terms of research output, alongside China and Japan. However, India's research performance on metrics such as citation and collaboration has been worrisome according to the report. Similar to other Asian countries in the G20, international collaboration is lower than the group average and growth is mostly driven by domestic research (73%). Additionally, Indonesia's share of domestic output is only 26%. The report found a common trend of higher category-normalised citation impact in countries with research output through international collaboration. Indian researchers published more than the global average on topics related to agricultural production, health and sustainable energy, medicines and vaccines for tuberculosis, and traditional knowledge; however, the output seemed to have a lower impact in most disciplines (including medicine), whereas Indonesia had a higher impact than the global average. Most south Asian countries depend on international collaborations for at least 50% of publications, and such collaborations seem to have higher citations. Collaborations between academic institutions and the private sector accounted for only 1.3% of south Asia's scholarly output, almost half of the global average.

International collaboration, as the report indicates, brings in diverse perspectives and seems to enhance the quality of research. However, the system of international collaboration is plagued with power imbalances and does not always play out to the advantage of local researchers. Current systems (including the role of donors, publishing journals, and working conditions of local researchers) are open to question as to whether they deliver the intended purpose.

Data indicates intraregional research collaboration among south Asian countries could yield substantial gains in a short period of time. The region has similar disease burden of communicable and non-communicable diseases. Collaborative research on various issues, such as sharing data on SARS-CoV-2 variants, could help in providing a more advanced response. Similarly, shared data could help to eliminate diseases, such as malaria. Increasing funding could advance research capacity in this region, but will need support by targeted policies designed to increase regional, international, public–private, and academia–industry collaborations.

At national level these collaboration efforts should be complemented by a diverse range of scholars in each country and include gender equity. In 2018, only 15% of researchers in India were working in the field of health and 25% of those health researchers were women. Despite a dedicated science and innovation policy, most countries in south Asia have inadequate instruments for quality research output. Formal institutional mechanisms, such as support to identify funding opportunities, managing programmes, public engagement, impact analysis, and ethics, are needed to support research management and academic leadership. To limit the declining number of researchers and tackle insufficient research output, tertiary education policies should be reviewed and enhanced, and brought in line with future research objectives including a dedicated focus on girls to pursue science, technology, engineering, and mathematics (STEM) subjects.

In the past few years, countries have aligned their research and development with the current challenges of COVID-19 and climate change in addition to the fast-approaching Sustainable Development Goals 2030. India's research and development response after the pandemic has focused on producing low-cost solutions predominantly of vaccines and generic versions of drugs and health-care such as low-cost lung ventilators. Pharmaceutical industries of Sri Lanka, Pakistan and Bangladesh has also boosted their research and development following the pandemic. The COVID-19 crisis has reminded us of the importance of collaboration in research and development. South Asian countries need to harness their research capacity collectively to address immediate priorities and contribute to regional and global science efforts. In its presiding role of G20, India should harness this opportunity to connect better with other research economies and play a bigger role in ensuring equity for researchers in international collaborations and contribute more to the ongoing research and development efforts in the south Asia region.

